# Acetylene Gas-Sensing Properties of Layer-by-Layer Self-Assembled Ag-Decorated Tin Dioxide/Graphene Nanocomposite Film

**DOI:** 10.3390/nano7090278

**Published:** 2017-09-18

**Authors:** Chuanxing Jiang, Dongzhi Zhang, Nailiang Yin, Yao Yao, Talgar Shaymurat, Xiaoyan Zhou

**Affiliations:** 1 College of Information and Control Engineering, China University of Petroleum (East China), Qingdao 266580, China; jiangchuanxing@126.com (C.J.); nailiang_yin@163.com (N.Y.); 2 College of Communication Engineering, Chengdu University of Information Technology, Chengdu 610225, China; yaoyao@cuit.edu.cn; 3 Key Laboratory of New Energy and Materials Research, Xinjiang Institute of Engineering, Urumqi 83000, China; talgar.shaymurat@vip.163.com; 4 College of Science, China University of Petroleum (East China), Qingdao 266580, China; zhouxiaoyan@upc.edu.cn

**Keywords:** graphene, layer-by-layer self-assembly, nanocomposite film, acetylene sensor

## Abstract

This paper demonstrates an acetylene gas sensor based on an Ag-decorated tin dioxide/reduced graphene oxide (Ag–SnO_2_/rGO) nanocomposite film, prepared by layer-by-layer (LbL) self-assembly technology. The as-prepared Ag–SnO_2_/rGO nanocomposite was characterized by scanning electron microscopy (SEM), transmission electron microscopy (TEM), X-ray diffraction (XRD) and Raman spectrum. The acetylene sensing properties were investigated using different working temperatures and gas concentrations. An optimal temperature of 90 °C was determined, and the Ag–SnO_2_/rGO nanocomposite sensor exhibited excellent sensing behaviors towards acetylene, in terms of response, repeatability, stability and response/recovery characteristics, which were superior to the pure SnO_2_ and SnO_2_/rGO film sensors. The sensing mechanism of the Ag–SnO_2_/rGO sensor was attributed to the synergistic effect of the ternary nanomaterials, and the heterojunctions created at the interfaces between SnO_2_ and rGO. This work indicates that the Ag–SnO_2_/rGO nanocomposite is a good candidate for constructing a low-temperature acetylene sensor.

## 1. Introduction

Acetylene (C_2_H_2_) is a colorless and highly combustible gaseous hydrocarbon, widely used as a fuel in many industrial fields, such as metal welding [1], polyacetylene preparation [2], lithium-ion batteries [3], and conductive plastic manufacturing [4]. However, acetylene is unstable and there is a huge potential risk of fire or explosive accidents during its compression and heat treatment, or due to leakage. Furthermore, the dissolved content of acetylene gas in power transformer oil is critical to the safety and reliability of the transformer system [5,6]. Therefore, reliable, economical and portable acetylene gas sensors are of great importance to many applications. In recent years, a lot of interest has been attracted surrounding the development of effective techniques and sensitive methods for acetylene gas detection, such as photoacoustic spectroscopy [7], optical fiber [6,8] and metal-oxide semiconductors (MOS) and nanomaterial-based sensors (i.e., PdO-decorated SnO_2_ [9], Au/multi-wall carbon nanotubes [10], Sm_2_O_3_-decorated SnO_2_ [11], Ag-loaded ZnO [12,13,14] and NiO/SnO_2_ heterostructures [15]). Among them, metal oxides have become important candidates for acetylene sensing due to their unique advantages—such as their small size and simplicity of integration—but they lack selectivity towards different gas species, and often require high operating temperatures and have high power consumption [16,17,18,19,20].

The state-of-the-art MOS-based acetylene sensor focuses on noble metal-metal oxide nanohybrids and heterometal oxide nanostructures. Zhang et al. reported on the hydrothermal synthesis of hierarchical nanoparticle-decorated ZnO microdisks for acetylene gas sensing at 420 °C [21]. Tamaekong et al. synthesized Pt/ZnO thick film, using the flame spray pyrolysis (FSP) technique, and a low detection limit for 50 ppm acetylene gas was obtained at an operating temperature of 300 °C [22]. Chen et al. synthesized Pd-doped SnO_2_ nanoparticles using a hydrothermal method for detecting acetylene gas dissolved in power transformer oil, indicating a sensor response of 7.22 for 100 ppm acetylene at 350 °C [23]. Zhou et al. fabricated a planar-type acetylene gas sensor based on Sm_2_O_3_-decorated SnO_2_ heterostructures, and showed that the optimum operating temperature of the sensor for 50 ppm of acetylene is 260 °C [11]. Uddin et al. developed a novel flexible acetylene gas sensor, consisting of Ag-loaded vertical ZnO nanorods, supported by a polyimide/ polytetrafluoroethylene (PI/PTFE) substrate, using a hydrothermal-radio frequency (RF) magnetron sputtering method and showed that its best sensing performance was at 200 °C [12]. As a two-dimensional nanomaterial, graphene has attracted much attention since its discovery, because of its excellent characteristics, such as its electrical, chemical and optical properties [24,25]. Graphene-based nanocomposites have been widely used in membrane science and technology [26,27]. Uddin et al. synthesized a ZnO/reduced graphene oxide (rGO) composite using a solvothermal method, which exhibited preferential detection of acetylene gas with good selectivity, long-term stability, and fast response/recovery times at 250 °C [28]. The presented progresses suggest that noble metal doping and graphene addition techniques are effective for lowering the operating temperature and improving the acetylene sensing performance of MOS-based sensors.

In this work, we fabricated a low-temperature acetylene gas sensor based on a layer-by-layer, self-assembled Ag–SnO_2_/rGO ternary nanocomposite film, for the first time. The as-prepared nanocomposite was characterized by scanning electron microscopy (SEM), transmission electron microscopy (TEM), X-ray diffraction (XRD) and Raman spectrum, which confirmed its successful formation and rationality. The acetylene sensing properties were investigated under different working temperatures and gas concentrations. An optimal temperature of 90 °C was determined, and the Ag–SnO_2_/rGO nanocomposite sensor exhibited excellent sensing behaviors towards acetylene in terms of response, repeatability, stability and response/recovery characteristics. The underlying sensing mechanism of the Ag–SnO_2_/rGO sensor was further discussed.

## 2. Materials and Methods

### 2.1. Materials

The high-purity graphene oxide (GO) nanosheets (>99%) were supplied by Chengdu Organic Chemicals Co. Ltd. (Chengdu, China). The GO used was graphene nanosheet, negatively decorated with oxygen functional groups and carboxylic groups, which were located at the sheet surface. The GO suspension was 0.25 wt % concentrated at pH 4.5. Tin chloride pentahydrate (SnCl_4_·5H_2_O) and hydrazine hydrate were obtained from Sinopharm Chemical Reagent Co. Ltd. (Shanghai, China). Polyelectrolytes used for layer-by-layer (LbL) assembly were 1.5 wt % poly(diallyldimethylammonium chloride) [PDDA (Sigma-Aldrich Co., Saint Louis, MO, USA), molecular weights (MW) of 200–350 K] and 0.3 wt % poly(sodium 4-styrenesulfonate) [PSS (Sigma-Aldrich Co., Saint Louis, MO, USA), MW of 70 K] with 0.5 M NaCl (West Long Chemical Co., Ltd., Guangdong, China) in both, to provide better surface coverage. All reagents were used without further purification.

### 2.2. Preparation of the Ag–SnO_2_/rGO Nanocomposite

**Figure 1:**
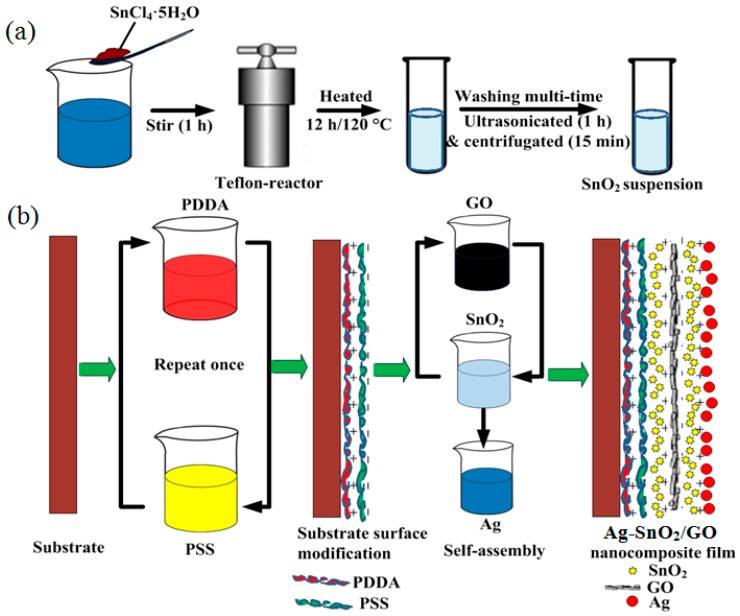
(**a**) Hydrothermal synthesis of SnO_2_ and (**b**) layer-by-layer self-assembly of the Ag–SnO_2_/GO nanocomposite film.

Figure 1a illustrates the hydrothermal synthesis of SnO_2_. Firstly, 24 mg of SnCl_4_·5H_2_O was dissolved in 30 mL of deionized water and stirred for 1 h. After that, the solution was hydrothermally treated at 120 °C for 12 h, and then washed with deionized water and ethanol several times. The SnO_2_ aqueous solution was obtained after being ultrasonicated for 1 h and centrifugated for 15 min. A substrate with interdigitated electrodes for resistive sensing and heating elements was fabricated. The Ag–SnO_2_/rGO nanocomposite was deposited on the sensing electrodes using the layer-by-layer (LbL) self-assembly technique, which is shown in Figure 1b. Two bi-layers of PDDA/PSS were firstly self-assembled as the precursor layer, followed by alternative immersion into SnO_2_, GO, SnO_2_ and Ag suspensions. The immersing time here used was 10 min for the polyelectrolytes and 15 min for the SnO_2_, GO and Ag suspensions. Intermediate rinsing with deionized water and drying with nitrogen gas were required after each monolayer assembly, to reinforce the interconnection between the layers. The film was formed due to the interaction of electrostatic forces between the positively and negatively charged nanoparticles. The first SnO_2_ layer (positively charged) was designed for the intermediate bonding between PSS (negatively charged) and GO (negatively charged). The Ag–SnO_2_/rGO nanocomposite sensor was obtained via the thermal reduction of GO into rGO at 220 °C for 5 h in an oven. Furthermore, the pure SnO_2_ and SnO_2_/rGO film sensor were fabricated to allow a comparison between the drop-casting and LbL self-assembly methods, respectively.

### 2.3. Instruments and Analysis

Surface microscopy of the Ag–SnO_2_/rGO sample was carried out with a scanning electron microscope (SEM, Hitachi S-4800, Tokyo, Japan). The X-ray diffraction (XRD) spectrum of the samples was examined with an X-ray diffractometer (Rigaku D/Max 2500PC, Tokyo, Japan) using Cu Kα radiation with a wavelength of 1.5418 Å. The lattice fringes of Ag–SnO_2_/rGO were inspected with a transmission electron microscope (FEI Tecnai G2 F20, Shanghai, China). The nanostructural and compositional features of the Ag–SnO_2_/rGO and SnO_2_/rGO samples were characterized by Raman spectra (RamLab-010, Horiba Jobin Yvon, Paris, France).

**Figure 2:**
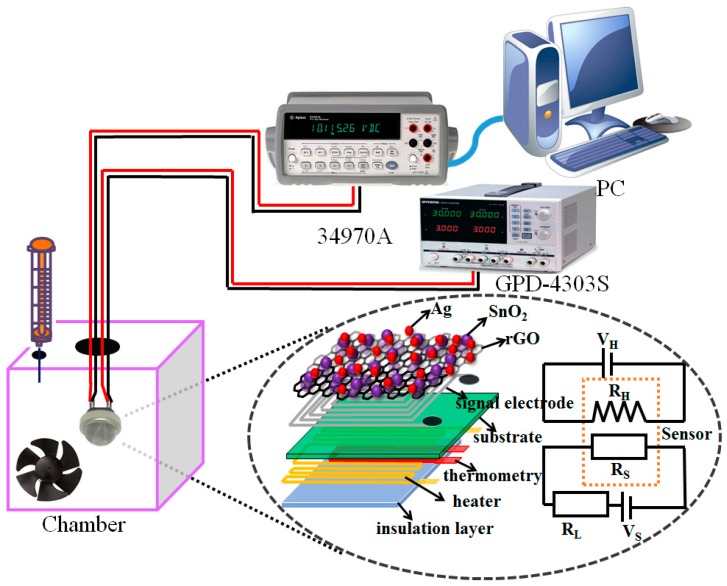
Schematic of the acetylene sensing experimental setup.

A schematic of the experimental setup for acetylene sensing is shown in Figure 2. The acetylene gas sensing properties were investigated by exposing the sensor to various concentrations of acetylene gas, and the desired gas concentration was obtained by injecting the required quantity of acetylene into a sealed chamber using a syringe. The working temperature for the sensor was adjusted through applying varying voltages to the heating electrodes with a power source (Gwinstek GPD-4303S, New Taipei, Taiwan). The heater resistor (*R*_H_), heating voltage (*V*_H_), sensor resistance (*R*_S_) and protection resistor (*R*_L_) made up the simplified circuit. The sensor resistance was recorded using a data logger (Agilent 34970A, San Jose, CA, USA), connected to a computer via a recommended standard (RS)-232 interface. The sensitivity of the sensor was defined as *S* = (*R*_0_ − *R*_g_)/*R*_0_ × 100%, where *R*_0_ and *R*_g_ were the sensor resistances in dry air and acetylene gas, respectively. The time taken by a sensor to achieve 90% of the total resistance change was defined as the response or recovery time. 

## 3. Results and Discussion

### 3.1. Sample Characterization

**Figure 3:**
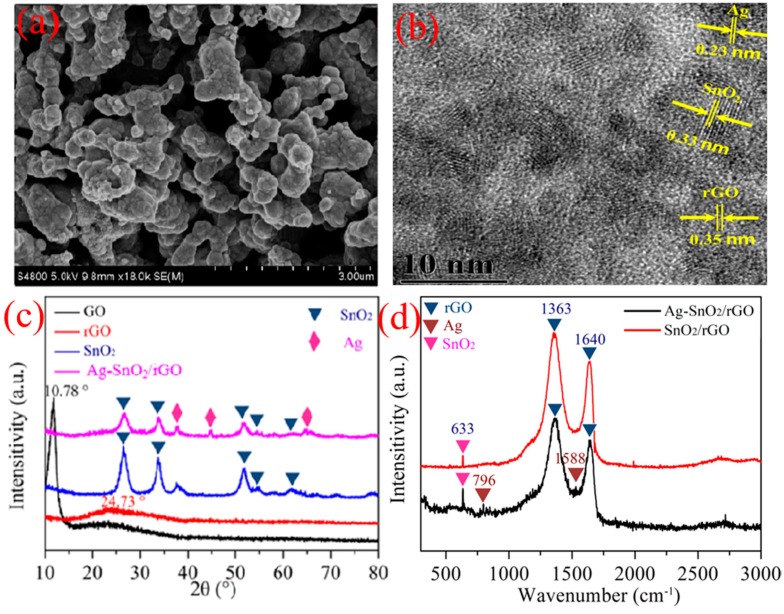
(**a**) SEM characterization of the Ag–SnO_2_/rGO nanocomposite, (**b**) TEM image of the Ag–SnO_2_/rGO nanocomposite, (**c**) XRD spectrum of GO, rGO, SnO_2_ and the Ag–SnO_2_/rGO nanocomposite, (**d**) Raman spectrum of the SnO_2_/rGO and Ag–SnO_2_/rGO nanocomposites.

Figure 3a shows the SEM image of the Ag–SnO_2_/rGO nanocomposite film. SnO_2_ microspheres and Ag nanoparticles (NPs) attached to the surface of rGO sheets are clearly observed. Figure 3b shows a high-resolution TEM image of the Ag–SnO_2_/rGO nanocomposite, and lattice fringe spacings of 0.35, 0.33 and 0.23 nm for rGO, SnO_2_ and Ag, respectively, are measured. Figure 3c indicates the XRD spectrum for the GO, rGO, SnO_2_ and Ag–SnO_2_/rGO nanocomposite films. Obvious peaks at 2*θ* angles of 10.78° and 24.7° are observed for the GO and thermally treated rGO, respectively, which is in agreement with previously published results, and further confirms the successful reduction of GO via thermal treatment [29,30,31]. The XRD spectrum of SnO_2_ indicates several peaks at 26.41°, 33.82°, 37.60°, 51.73° and 65.68°, indexed to the (110), (101), (200), (211) and (301) planes of rutile SnO_2_, which is in accordance with the data from JCPDS Card no. 41-1445 [32], and confirms the successful formation of SnO_2_ nanocrystals. Apart from the characteristic peaks attributed to SnO_2_, the XRD spectrum of the Ag–SnO_2_/rGO nanocomposite exhibited distinct peaks at 38.10°, 44.37° and 64.17°, which indexed to the (111), (200) and (220) planes of Ag crystallines, respectively [33]. However, the broad peak of rGO is not obvious in the XRD pattern of the Ag–SnO_2_/rGO nanocomposite, probably because the weak peak of rGO is swamped by the high intensity peak of the SnO_2_ at the 2*θ* angle of 26.41° [34,35]. Figure 3d shows the Raman spectrum of the SnO_2_/rGO and Ag–SnO_2_/rGO nanocomposites. The peaks located at 633 cm^−1^ in the SnO_2_/rGO and Ag–SnO_2_/rGO nanocomposites are typical Raman peaks of SnO_2_. The weak peaks located at 796 and 1588 cm^−1^ for the two samples are attributed to the Ag NPs. The peaks located at 1363 and 1640 cm^−1^ are attributed to defects and disorder in the graphite layer.

### 3.2. Acetylene Sensing Properties

**Figure 4:**
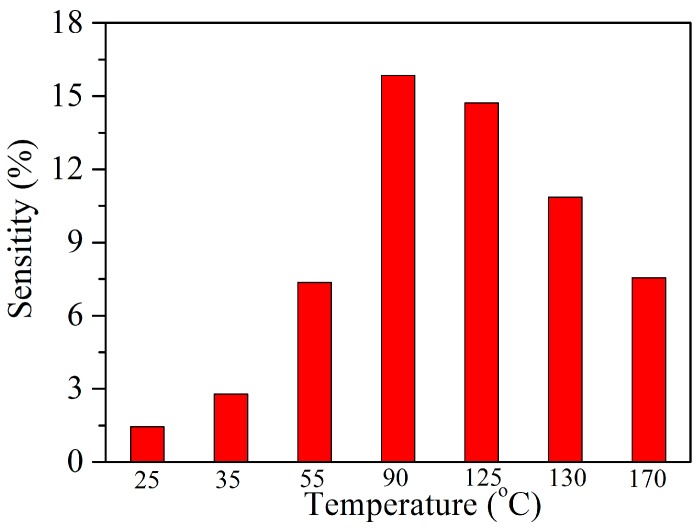
The sensitivity of the Ag–SnO_2_/rGO nanocomposite sensor to 100 ppm acetylene under different operating temperatures.

Figure 4 shows the sensitivity of the Ag–SnO_2_/rGO nanocomposite sensor to 100 ppm acetylene under operating temperatures of 25 to 170 °C. The sensor sensitivity increases and reaches its highest value at 90 °C, and then decreases with any further increase in temperature. This can be explained by the fact that the appropriate operating temperature improves sensor sensitivity, but higher temperatures lower the binding energies of gas molecules and sensing film. An optimal temperature of 90 °C was determined. Therefore, the operating temperature of 90 °C was selected for the sensor in the following experiments.

**Figure 5:**
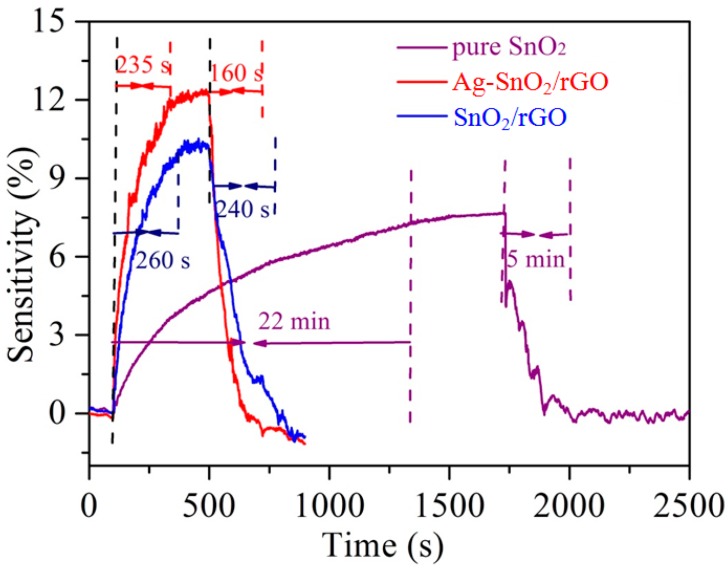
The sensitivity of pure SnO_2_, SnO_2_/rGO and Ag–SnO_2_/rGO film sensors to 50 ppm acetylene at 90 °C.

Figure 5 shows the sensitivity of pure SnO_2_, SnO_2_/rGO and Ag–SnO_2_/rGO film sensors to 50 ppm acetylene at 90 °C. We found that the Ag–SnO_2_/rGO film sensor yielded the highest sensitivity among the three sensors. Moreover, the Ag–SnO_2_/rGO film sensor showed a shorter response/recovery time than the other two sensors. A response time and recovery time of 235 and 160 s, respectively, were observed for the Ag–SnO_2_/rGO film sensor upon exposure to 50 ppm acetylene.

**Figure 6:**
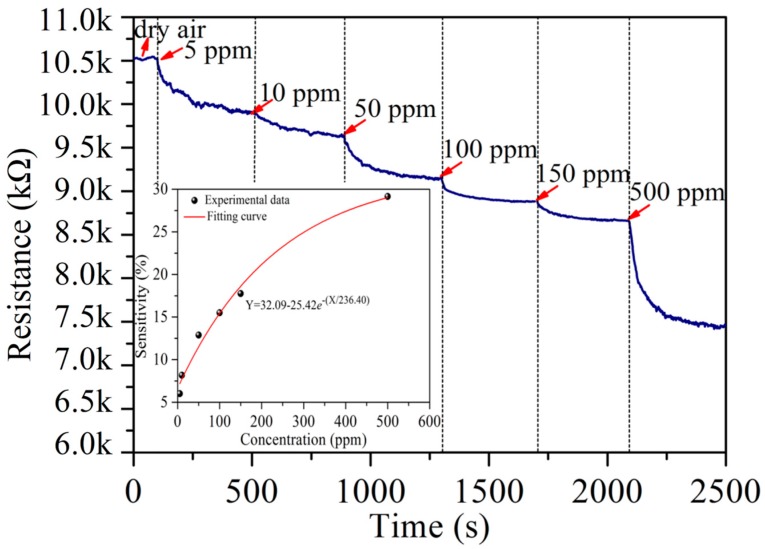
Resistance measurement of the Ag–SnO_2_/rGO nanocomposite film sensor upon exposure to acetylene gas with cumulated concentrations at 90 °C.

Figure 6 shows the resistance measurements for the Ag–SnO_2_/rGO nanocomposite film sensor upon exposure to acetylene gas, of cumulative concentrations, at 90 °C. The test was performed by exposing the sensor to 5, 10, 50, 100, 150 and 500 ppm of acetylene. The resistance of the Ag–SnO_2_/rGO nanocomposite sensor decreased with increasing concentrations of acetylene, indicating the n-type semiconductor-like behavior of Ag–SnO_2_/rGO to acetylene (reducing gas). The inset of Figure 6 plots the fitted function of sensor sensitivity (*Y*) and acetylene concentration (*X*) as *Y* = 32.09 − 25.42*e*^−*X*/236.4^.

**Figure 7:**
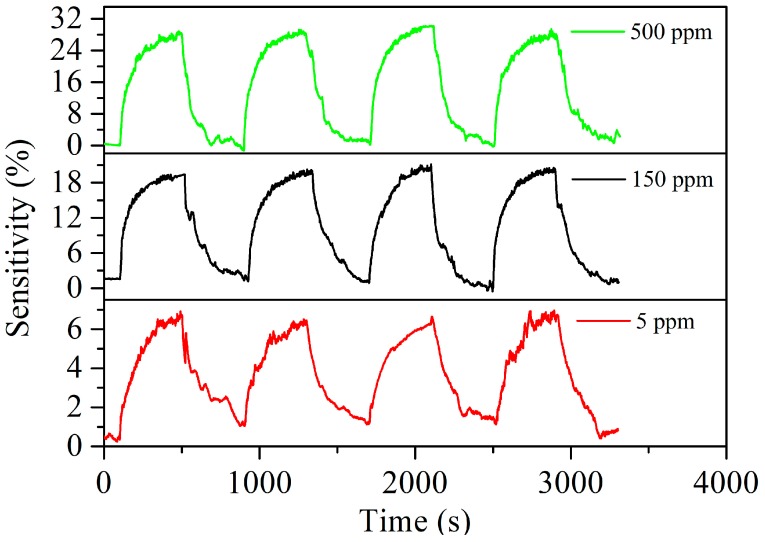
Repeatability of the Ag–SnO_2_/rGO nanocomposite film sensor toward acetylene concentrations of 5, 150 and 500 ppm at 90 °C.

**Figure 8:**
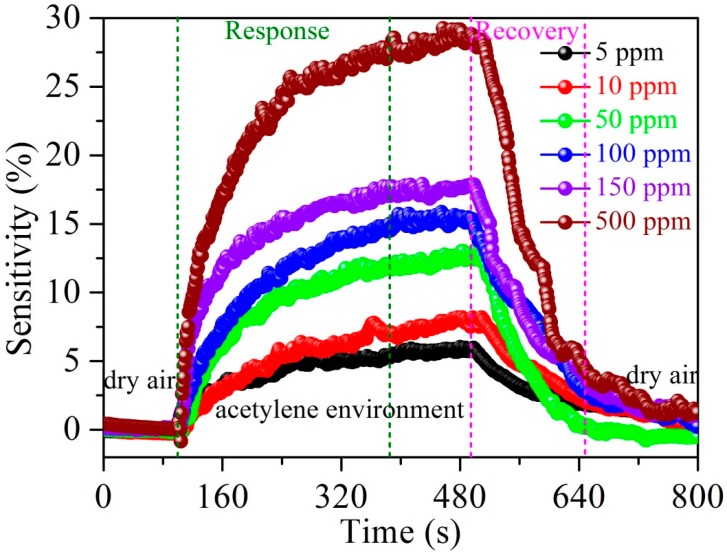
Typical response and recovery curves of the Ag–SnO_2_/rGO nanocomposite film sensor to acetylene pulses between 0 and 500 ppm.

Figure 7 shows the repeatability of the Ag–SnO_2_/rGO film sensor with concentrations of 5, 150 and 500 ppm acetylene at 90 °C. There were no significant changes in sensor sensitivity during the repeated exposure/recovery cycles, indicating an acceptable repeatability for acetylene sensing. Figure 8 demonstrates the typical response and recovery curves of the Ag–SnO_2_/rGO film sensor towards an acetylene pulse, at concentrations between 0 and 500 ppm, exhibiting good response/recovery behavior. 

**Figure 9:**
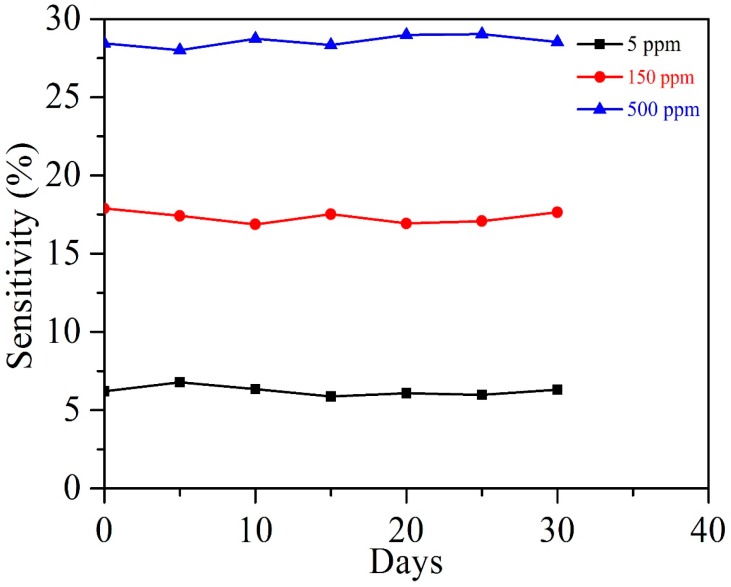
Long-term stability of the Ag–SnO_2_/rGO nanocomposite film sensor, measured every five days for over 30 days.

**Figure 10:**
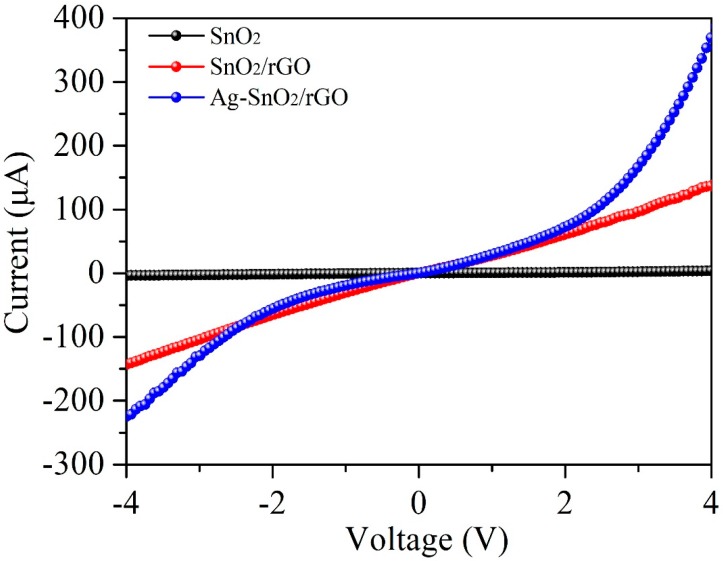
The measured I–V curves of the SnO_2_, SnO_2_/rGO and Ag–SnO_2_/rGO films.

Figure 9 shows the response of the Ag–SnO_2_/rGO nanocomposite film sensor to concentrations of 5, 150, and 500 ppm acetylene gas, measured every five days for over 30 days. It was clearly shown that the sensor response does not vary significantly with time, confirming that the Ag–SnO_2_/rGO nanocomposite film sensor has good long-term stability. Figure 10 shows the experimental current–voltage (I–V) curves for the SnO_2_, SnO_2_/rGO and Ag–SnO_2_/rGO film devices, measured by applying voltages from −4 to 4 V. It is clearly shown that the measurement results indicate good Ohmic contact to n-type semiconductors for the three devices. A larger current passes through the Ag–SnO_2_/rGO sensor than that of the SnO_2_/rGO and SnO_2_ sensors. This is because the doping of Ag and rGO results in a decrease in the resistance of the Ag–SnO_2_/rGO film. Table 1 presents the performance of the proposed acetylene gas sensor in comparison with previous reported works [12,13,15,23,36,37,38]. The working temperature and responses for the prepared sensor are comparable to metal oxide-based sensors made by hydrothermal-RF magnetron sputtering, hydrothermal, electrospinning, spin-coating and sol-gel methods. The presented Ag–SnO_2_/rGO film sensor exhibited a much higher response and a lower working temperature than that of its MOS-based counterparts. 

### 3.3. Acetylene-Sensing Mechanism

**Figure 11:**
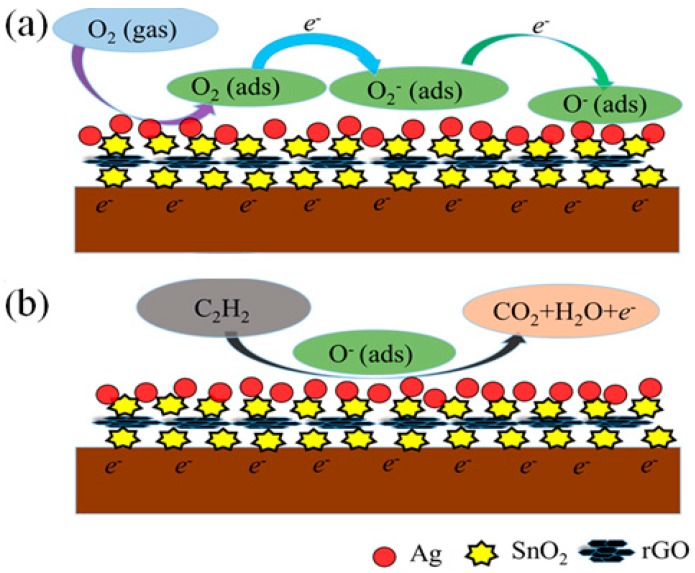
The sensing mechanism of the Ag–SnO_2_/rGO nanocomposite film in (**a**) air and (**b**) acetylene gas.

The Ag–SnO_2_/rGO ternary nanocomposite film demonstrated excellent sensing properties towards acetylene gas at low temperatures. Its sensitive mechanism can be attributed to the synergistic effect of the ternary hybrids and the created potential barrier. Pristine SnO_2_ is an n-type semiconductor and electrons are majority carriers. Graphene, as one of the emerging 2D nanomaterials, has a unique layered structure, a large surface area-to-volume ratio and excellent electrical properties, which greatly facilitate the absorption and diffusion of acetylene gas molecules. Figure 11 shows the sensing mechanism of the Ag–SnO_2_/rGO nanocomposite film in air and acetylene gas. The oxygen molecules adsorbed on the conduction band of n-type SnO_2_ are ionized to oxygen negative ions through the trapping of free electrons from the surface of the SnO_2_ [39]. When the sensor is exposed to acetylene gas, the adsorbed acetylene interacts with the oxygen’s negative ions and produces carbon dioxide, water molecules and free electrons. The reaction is expressed as C_2_H_2_ (ads) + O^−^ (ads) → CO_2_ + H_2_O + e^−^, which leads to a decrease in the sensor’s resistance [14,28]. 

**Figure 12:**
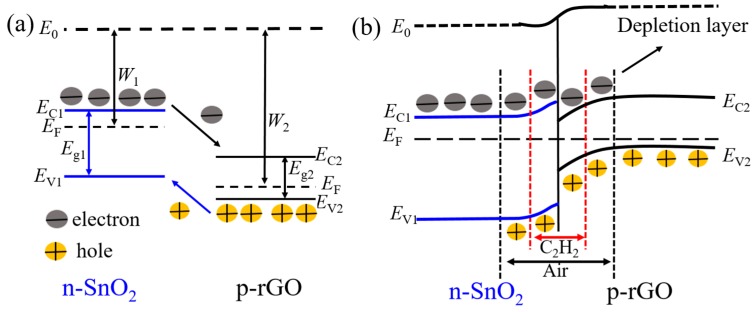
Schematic of the energy band diagram and the variation in depletion layer thickness for the SnO_2_/rGO heterojunction. (*E*_C_, conductor band; *E*_g_, band gap; *E*_V_, valence band; *E*_F_, Fermi level; W, work function)

The formation of a heterojunction at the interface between rGO and SnO_2_ is beneficial, because it enhances the acetylene-sensing properties. Figure 12a shows a schematic of an energy band diagram of the SnO_2_/rGO heterojunction. The band-gaps for n-type SnO_2_ and p-type rGO are 3.6 and 0.4 eV, respectively [34] and their work functions are 4.5 and 5.1 eV for SnO_2_ and rGO, respectively [40,41]. Because the Fermi energies are not at the same level and the rGO has a higher work function, when SnO_2_ and rGO come into contact with each other, electrons transfer from SnO_2_ to rGO, and holes flow in the opposite direction until a dynamic equilibrium state is reached, and thus a depletion layer is formed at the interface [42]. Figure 12b shows the variation of depletion layer thickness for the SnO_2_/rGO heterojunction. When the sensor was exposed to acetylene gas, the interaction between adsorbed O^−^ and acetylene molecules released free electrons, with the released free electrons increasing the n-type doping of SnO_2_. Higher SnO_2_ doping results in a reduced depletion layer in SnO_2_, thereby decreasing the sensor resistance during acetylene gas exposure. 

The Ag NPs in the nanocomposite have a positive effect on electronic sensitization during gas adsorption [43,44]. A potential barrier can be created at the contact interface between SnO_2_ and Ag, which is beneficial to the enhancement of gas-sensing. Electrons transfer from SnO_2_ to Ag NPs and form a highly-resistive “barrier layer” in air, and Ag NPs become centers for electron accumulation. When the sensor is exposed to acetylene gas, electrons transfer from acetylene gas to the Ag NPs and then to SnO_2_; the highly-resistive “barrier layer” is transformed into a highly-conductive “anti-barrier layer”, which improves the electron mobility and sensing performance of the Ag–SnO_2_/rGO nanocomposite sensor.

## 4. Conclusions

In this work, an acetylene gas sensor, based on Ag–SnO_2_/rGO nanocomposite film was fabricated by layer-by-layer self-assembly technology. Successful preparation of the Ag–SnO_2_/rGO nanocomposite was characterized and confirmed by means of SEM, TEM, XRD and Raman spectrum. The Ag–SnO_2_/rGO nanocomposite sensor exhibited excellent sensing behaviors towards acetylene in terms of response, repeatability, stability and response/recovery characteristics at an optimal temperature of 90 °C, which are superior to pure SnO_2_ and SnO_2_/rGO film sensors. The underlying sensing mechanism of the Ag–SnO_2_/rGO sensor was explored. This work provides guidance for an acetylene sensor based on Ag–SnO_2_/rGO ternary nanocomposites.

## Figures and Tables

**Table 1:** Performance of the presented sensor in this work compared with previous works.

Sensing Material	Fabrication Method	Work Temperature	Response	Reference
Ag–SnO_2_/rGO	Layer-by-layer self-assembly	90 °C	15.8 @100 ppm	This paper
Ag–ZnO nanorods	Hydrothermal-radio frequency (RF) magnetron sputtering	200 °C	27.2 @1000 ppm	[12]
Ag-hierarchical ZnO	Hydrothermal method	200 °C	1.92 @1000 ppm	[13]
NiO/SnO_2_	Hydrothermal method	206 °C	13.8 @100 ppm	[15]
PdO-SnO_2_	Hydrothermal method	350 °C	7.22 @100 ppm	[23]
Ni-ZnO	Electrospinning method	250 °C	17 @2000 ppm	[36]
SnO_2_	Spin-coating method	300 °C	6.3 @10000 ppm	[37]
Sm_2_O_3_/SnO_2_	Sol-gel method	180 °C	63.8 @1000 ppm	[38]
